# 肺小结节术前CT引导下微弹簧圈与硬化剂定位的对比分析

**DOI:** 10.3779/j.issn.1009-3419.2020.102.07

**Published:** 2020-06-20

**Authors:** 杲 吴, 显宁 吴, 美青 徐

**Affiliations:** 230001 合肥，中国科学技术大学附属第一医院（安徽省立医院）胸外科 Department of Thoracic Surgery, the First Affiliated Hospital of University of Science and Technology of China (Anhui Provincial Hospital), Hefei 230001, China

**Keywords:** 肺小结节, 定位, 微弹簧圈, 硬化剂, Solitary pulmonary nodule, Localization, Microcoil, Sclerosing agent

## Abstract

**背景与目的:**

肺内小结节往往难以进行胸腔镜术中定位，需要术前计算机断层扫描（computed tomography, CT）引导经皮穿刺定位辅助，本研究旨在比较两种不同定位材料即微弹簧圈和硬化剂（聚桂醇）定位的效果及相关并发症，评价优劣。

**方法:**

回顾性分析术前CT引导经皮穿刺定位患者371例，根据采用的不同定位材料分为：微弹簧圈组（microcoil group）167例，定位结节196枚、硬化剂组（sclerosing agent group）204例，定位结节239枚。统计分析两组定位效果、并发症、病理资料及手术方案等相关资料。

**结果:**

微弹簧圈组定位失败率（2.4%）高于硬化剂组（0.5%）（*P*=0.011），硬化剂定位耗时明显短于微弹簧圈组[（18.78±6.91）min *vs*（11.99±3.77）min, *P*=0.000]，但微弹簧圈组较硬化剂组选择定位结节与胸膜间距离更深[（9.59±8.62）mm *vs*（8.13±6.49）mm, *P*=0.002]。总体并发症上微弹簧圈组显著高于硬化剂组（*P*=0.000），其中以气胸为主，通过相关危险因素分析提示不同定位方法是独立危险因子。手术方式以楔形切除为主，病理结果以非浸润性病变为主。

**结论:**

微弹簧圈与硬化剂均是良好的术前定位材料，聚桂醇硬化剂较之微弹簧圈定位失败率更低、并发症更少，定位时长更短，操作简便且费用低廉，值得推广。

## Abstract (en)

**Background and objective:**

Small pulmonary nodules are usually difficult to identify during thoraco-scopic resection, and preoperative computed tomography (CT)-guided percutaneous puncture assisted localization can be helpful. The purpose of this study is to compare the localization effect and complication rates of two different methods by microcoil placement and sclerosing agent injection (Lauromacrogol).

**Methods:**

A retrospective analysis of the clinical data of 371 patients with preoperative pulmonary nodules percutane us puncture localization was performed. According to the use of different materials, they were divided into the microcoil group (167 cases with 196 localized nodules) and the sclerosing agent group (204 cases with 239 localized nodules). The localization effect, complication, pathological results and operation relates data were statistically analyzed.

**Results:**

The localization failure rate (2.4%) was higher in the microcoil group than in the sclerosing agent group (0.5%) (*P*=0.011), and the localization time of sclerosing agent group was significantly shorter than the microcoil group (18.78±6.91) min *vs* (11.99±3.77) min, *P*=0.000, but the distance between the selected localized nodules and the pleura was deeper in the microcoil group than in the sclerosing agent group (9.59±8.62) mm *vs* (8.13±6.49)mm, *P*=0.002. The overall complications in the microcoil group were significantly higher than those in the sclerosing agent group (*P*=0.000), in which pneumothorax was the most common. Through the analysis of related risk factors, we revealed that different positioning methods was independent risk factors. Wedge resection was the main type of surgical method and non-invasive carcinomas were the majority of postoperative pathological results.

**Conclusion:**

Our study suggests that both microcoil placement and sclerosing agent injection are suitable for preoperative pulmonary nodule localization equivalently, however, compared with microcoils placement, injection of lauromacrogol, the sclerosing agent, had lower failure rate, less complications, shorter localization time and it is worthy of promotion also by easy operation and low cost.

近年来，肺小结节（small pulmonary nodules, SPNs）的检出率日益增高，对肺小结节的影像学形态的分析和性质预测越来越深入且准确。对于可疑恶性的结节，尤其是亚实性结节，难以通过常规气管镜活检及经皮穿刺活检取的病理诊断。因此，通过电视辅助胸腔镜手术（video-assisted thoracoscopic, VATS）完成其诊断和治疗成为首选的方案^[1]^，然而大部分胸膜下结节难以看见，术中触诊率低，会导致被迫中转开胸甚至手术失败^[2]^。因此，肺小结节的术前定位尤为重要，我科自2015年开展计算机断层扫描（computed tomography, CT）引导下经皮穿刺辅助定位，前后采用两种不同材料即微弹簧圈和硬化剂（聚桂醇注射液）定位，均取得良好定位效果，本研究对比两种不同定位材料的应用效果及相关并发症，分析两者利弊及应用价值，现报道如下。

## 1 材料与方法

### 1.1 临床资料

收集我科自2016年1月-2018年11月间肺内小结节采用CT引导下经穿刺辅助定位的患者共371例，术前定位结节共435枚，其中定位单个结节320例，定位多个结节51例（其中位于一侧肺内同一肺叶8例，位于一侧肺内不同肺叶24例，位于两侧肺内19例），采用微弹簧圈定位167例，定位结节196枚，硬化剂定位204例，定位结节239枚，两组一般资料见表 1。

**1:** 两组一般资料 Clinical characteristics of the two groups

General data	Microcoil group (*n*=167)	Sclerosing agent group (*n*=204)
Gender		
Male	77 (46.1%)	81 (39.7%)
Female	90 (53.9%)	123 (60.3%)
Age（Mean±SD, yr）	55.70±11.33	55.03±11.22
Number of localized nodules		
Single	145 (86.8%)	175 (85.8%)
Multiple	22 (13.2%)	29 (14.2%)
Density of nodules		
GGO	88 (44.9%)	103 (43.1%)
Partly-solid GGO	48 (24.5%)	73 (30.5%)
Solid nodule	60 (30.6%)	63 (26.4%)
Diameter of nodules (mm)	9.14±4.25	9.20±4.34
Nodules location		
Right upper lobe	84 (42.9%)	88 (36.8%)
Right middle lobe	12 (6.1%)	11 (4.6%)
Right low lobe	37 (18.9%)	43 (18.0%)
Left upper lobe	40 (20.4%)	59 (24.7%)
Left low lobe	23 (11.7%)	38 (15.9%)
GGO: ground-glass opacity.		

### 1.2 纳入与排除标准

纳入标准：①高分辨率CT肺窗上直径 < 3 cm的纯磨玻璃样结节或实性成分 < 50%的亚实性结节，不伴有脏层胸膜皱缩；②高分辨率CT肺窗上直径≤1 cm，距离肺脏层胸膜≥1 cm的实性结节；③定位影像资料完整者。排除标准：①直径 < 4 mm的肺内单发孤立性结节不予以定位及切除；②定位影像学资料不全者。

### 1.3 设备与材料

CT设备：GE lightspeed4多排螺旋CT扫描仪，CT定位标记金属丝，PTC穿刺针（21 G，美国巴德），2%利多卡因，5 mL注射器，弹簧圈：美国COOK MWCE-18-14-8-NESTER栓塞弹簧圈，长度140 mm，直径0.018英寸，硬化剂：聚桂醇注射液，陕西天宇制药有限公司生产（国药准字H20080445），规格10 mL:100 mg。

### 1.4 定位方法

微弹簧圈组：术前1 d或手术当日根据术前结节位置行局部低剂量多层面CT扫描，并预先根据结节位置选择合适穿刺体位（遵循“垂直原则”即进针点尽量与结节所在肺叶胸膜面垂直），紧贴患者体表纵向放置CT定位标记金属丝，CT扫描定位结节最佳穿刺路径，确定体表穿刺点后测量穿刺角度及进针深度。穿刺点消毒局部麻醉后，在患者平静呼吸状态下按预定进针路径插入穿刺针，穿至邻近胸膜处暂停，再次CT扫描确定进针路径有无偏移，如有偏移则行局部调整，如确认路径准确，则将穿刺针快速插入肺内至预先设定深度，拔出穿刺针针芯，将弹簧圈推送杆顺穿刺针外鞘送达结节内或其边缘，先推出一小部分弹簧圈，预算针尖与脏层胸膜间距离，小心撤出穿刺针外鞘至胸膜面，再推出全部弹簧圈，最后CT扫描确认弹簧圈位置，有无脱钩及穿刺后气胸、血胸、穿刺道肺内血肿形成等，穿刺点包扎。留取穿刺定位过程CT影像资料供手术参考。

硬化剂组：前期患者准备、CT扫描定位结节穿刺路径、PTC进针方式与微弹簧圈组相同，在穿刺针到达预定位置后，拔出穿刺针针芯，将预先准备的5 mL注射器抽取2 mL-3 mL聚桂醇注射液接穿刺针外鞘，回抽确认有气体后缓慢注入药物，预计针尖与脏层胸膜间距离，一边注射一边退针，直至完全退出胸腔，再次CT扫描确认穿刺部位有无标记物显影及穿刺后气胸、血胸、肺内血肿形成等，穿刺点包扎。留取穿刺定位过程CT影像资料供手术参考。

### 1.5 手术方法

双腔气管插管全麻后，取键侧卧位，行单孔胸腔镜（切口位于腋中线与腋前线之间第5肋间，长3 cm-4 cm）或单操作孔胸腔镜（操作孔位于腋前线第4或第5肋间，长3 cm-4 cm，镜孔位于腋中线第7或第8肋间，长1 cm-1.5 cm），逐层切开，进胸探查，如有胸腔粘连，先电钩仔细分离，探查穿刺定位点，微弹簧圈组可见弹簧圈尾盘于胸膜表面，硬化剂组可见定位点局部脏层胸膜血肿样结节形成，卵圆钳滑动触诊结节定位处有弹跳感，即为定位成功。根据术前影像学判断设计手术切除方案，浅表结节可直接置入胸腔镜下直线切割缝合器行大楔形切除，确保切缘距离结节≥2 cm或 > 结节直径的1.5倍。结节位置较深者可行相应肺段切除或肺叶切除，标本离体后送检术中冰冻病理检查，如报告为浸润性腺癌，改行肺叶切除并行标准肺门纵隔淋巴结清扫。

### 1.6 统计学方法

采用SPSS 17.0进行统计学处理，计量资料使用均数±标准差（Mean±SD）表示，计数资料行*χ*^2^检验，计量资料符合正态分布的数据行*t*检验，采用二次元*Logistic*回归分析总体并发症的相关危险因素（包括定位方式、年龄、性别、病灶位置、结节与胸膜距离及胸膜穿刺点与结节间距离），*P* < 0.05为差异有统计学意义。

## 2 结果

### 2.1 一般资料

对比两组临床资料，微弹簧圈组167例中男性77例，女性90例，年龄21岁-82岁，平均年龄（55.70±11.33）岁，硬化剂组204例中男性81例，女性123例，年龄28岁-79岁，平均年龄（55.03±11.22）岁，年龄及性别比较无统计学差异。微弹簧圈组患者中诊断为肺多发病变共37例，最终定位多个结节22例，硬化剂组患者中诊断为肺多发病变共49例，最终定位多个结节29例。微弹簧圈组结节直径2 mm-27 mm，平均直径（9.14±4.25）mm，硬化剂组结节直径2 mm-26 mm，平均直径（9.20±4.34）mm。两组病例中结节位于右上肺叶居多（微弹簧圈组42.9%，硬化剂组36.8%），影像学上表现为GGO的比例较高（微弹簧圈组44.9%，硬化剂组43.1%），无论定位结节个数比例、结节直径、结节位置及结节密度，两组间均无统计学差异。

### 2.2 定位效果与并发症

微弹簧圈组中因术中发现弹簧圈脱钩4例，视为定位失败，定位失败率2.4%，硬化剂组中因硬化剂注射在壁层胸膜，肺表面未见任何标记1例，视为定位失败，定位失败率0.5%，比较两组存在统计学差别（*P* < 0.05）。微弹簧圈组定位时长7 min-47 min，平均时长（18.78±6.91）min，硬化剂组定位时长7 min-32 min，平均时长（11.99±3.77）min，两组比较有统计学差异（*P* < 0.01）。通过分别测量结节中心与邻近胸膜最短垂直距离及相应穿刺路径上穿刺针进入胸膜点的距离，统计微弹簧圈组结节距胸膜的平均距离为（9.59±8.62）mm，结节距胸膜穿刺点的平均距离为（14.34±10.02）mm，硬化剂组结节距胸膜的平均距离为（8.13±6.49）mm，结节距胸膜穿刺点的平均距离为（13.54±9.17）mm，微弹簧圈组中选择定位的结节较深（*P* < 0.05），但穿刺入路的选择上两组并无统计学差别。并发症方面，穿刺定位后发生气胸最为常见，总体气胸发生比例为微弹簧圈组22.2%、硬化剂组4.4%，后者明显少于前者。绝大部分为无症状的极少量气胸，微弹簧圈组中出现1例中等量气胸，术前即行胸腔闭式引流术减压，微弹簧圈组发生少量血胸3例（1.8%），定位后CT影像可见穿刺点附近气胸伴有少量积液影，术前未作特殊处理，术中探查见胸腔内血性胸液，弹簧圈尾附着凝血纤维素得以证实。另有1例63岁女性患者在放置弹簧圈过程中出现明显胸痛，考虑胸膜反应，终止操作后症状自行缓解。两组发生单纯少量咯血各3例。此外，我们观察到微弹簧圈组穿刺定位后出现针道周围肺泡内血肿12例（7.2%），而硬化剂组出现明显肺泡内血肿仅4例（2.0%），硬化剂组明显少于弹簧圈组。所有病例定位期间均未发生空气栓塞、大咯血等严重并发症及死亡。见表 2。

**2:** 两组定位资料及并发症统计表 Location data and complication statistics of the two groups

Variables	Microcoil group (*n*=167)	Sclerosing agent group (*n*=204)	*P*
Distance from nodules to pleura (Mean±SD, mm)	9.59±8.62	8.13±6.49	0.002
Distance from puncture point to nodules (Mean±SD, mm)	14.34±10.02	13.54±9.17	0.232
Average procedural time (Mean±SD, min)	18.78±6.91	11.99±3.77	0.000
Complication			
Pneumothorax	37 (22.2%)	9 (4.4%)	0.000
Hemoptysis	3 (1.8%)	3 (1.5%)	
Intralveolar hematoma	12 (7.2%)	4 (2.0%)	
Hemothorax	3 (1.8%)	0 (0.0%)	
Chest pain	1 (0.6%)	0 (0.0%)	
Failure	4 (2.4%)	1 (0.5%)	0.011
The patients with mutiple nodules with each localization procedure counted as a separate procedure.			

### 2.3 总体并发症的相关因素分析

在对总体并发症的相关危险因素（包括定位方式、年龄、性别、病灶位置、结节与胸膜距离及胸膜穿刺点与结节间距离）进行二次元*Logistic*回归分析时，我们将年龄按照世界卫生组织（World Health Organization, WHO）年龄划分标准分为44岁以下青年组、45岁-59岁中年组、60岁-74岁年轻老年组及75岁以上老年组。将结节与胸膜距离及穿刺点与结节间距离按 < 20 mm、20 mm-39 mm、≥40 mm三组进行重新编辑分类变量，分析结果提示定位方法是总体并发症的独立危险因子（*P* < 0.01）。此外穿刺点与结节间距离与总体并发症成正相关，但本研究中*P* > 0.05，并无统计学意义。见表 3。

**3:** 总体并发症的相关因素分析 Analysis of related factors of overall complications

		B	S.E	Wald	df	Sig.	Exp (B)
Step 1	Age	-0.239	0.178	1.804	1	0.179	0.788
	Distance from nodules to pleura	0.385	0.479	0.645	1	0.422	1.469
	Distance from puncture point to nodules	-0.593	0.308	3.705	1	0.054	0.553
	Nodules location	-0.068	0.092	0.550	1	0.458	0.934
	Location method	1.725	0.309	31.058	1	0.000	5.611
	gender	0.299	0.275	1.184	1	0.276	1.348
	Constant	-0.205	0.905	0.051	1	0.821	0.815
Variables entered in step 1: age, distance between nodules and pleura, distance between puncture points and nodules, nodules location, location method and gender.							

### 2.4 手术方案与术后病理

两组病例定位完成后当日或次日完成手术，微弹簧圈组结节切除成功率为100%，硬化剂组中有1例双肺转移瘤双侧定位后仅行单侧（右肺）结节切除，最终结节切除成功率为99.5%。两组均无因定位失败而中转开胸者，微弹簧圈组定位失败的4例患者在术中通过找寻脏层胸膜穿刺针眼完成切除，硬化剂组定位失败的1例患者根据术前影像判断完成结节所在肺段切除。本研究中楔形切除为主要手术方式，微弹簧圈组133例（79.6%），硬化剂组176例（86.3%），硬化剂组中出现全肺切除1例，系左下肺前基底段5 mm小结节合并左上肺上舌段近肺门部肉芽肿性炎（术前未证实），术中肺门结构解剖困难，改行全肺切除。术中快速病理结果以原位腺癌居多，微弹簧圈组71例（36.2%），硬化剂组72例（30.1%）。微弹簧圈组中微浸润腺癌28例（14.3%），腺癌11例（5.6%），硬化剂组中微浸润腺癌42例（17.6%），腺癌27例（11.3%），硬化剂组比例略高。但微弹簧圈组中非典型腺瘤样增生32例（16.3%），硬化剂组中非典型腺瘤样增生25例（10.5%），微弹簧圈组比例略高。两组病例中病理诊断为良性病变的比例差别较小，但硬化剂组中有6例少见肿瘤病例，分别是硬化性肺泡细胞瘤1例、梭形细胞瘤1例、类癌1例、纤毛粘液性结节性乳头性肿瘤1例、炎性肌纤维母细胞瘤1例、腺纤维瘤1例。见表 4。

**4:** 两组手术与病理资料 Surgcial and pathological parameters of the two groups

Variables	Microcoil group (*n*=167)	Sclerosing agent group (*n*=204)
Surgical procedures		
Wedge resection	133 (67.9%)	176 (73.6%)
Segmentectomy	39 (19.9%)	25 (10.5%)
Lobectomy	24 (12.2%)	36 (15.1%)
Pneumonectomy	0 (0.0%)	1 (0.4%)
None	0 (0.0%)	1 (0.4%)
Pathological pattern		
AIS	71 (36.2%)	72 (30.3%)
MIA	28 (14.3%)	42 (17.6%)
A	11 (5.6%)	27 (11.3%)
AAH	32 (16.3%)	25 (10.5%)
IL	33 (16.8%)	36 (15.1%)
Hamartoma	5 (2.6%)	7 (3.0%)
Metastatic tumor	4 (2.1%)	4 (1.7%)
Benign lesion	10 (5.1%)	13 (5.5%)
Lymph node	2 (1.0%)	6 (2.5%)
Others	0 (0.0%)	6 (2.5%)
AIS: adenocarcinoma in situ; MIA: minimally invasive adenocarcinoma; A: adenocarcinoma; AAH: atypical adenomatous hyperplasia; IL: inflammatory lesion; Others: rare tumors that are difficult to classify. The patients with mutiple nodules with each surgical procedure counted as a separate procedure.		

## 3 讨论

肺小结节的检出率逐年增高，但胸腔镜微创技术对于胸膜下小结节，尤其是磨玻璃样结节的处理仍然有限，术前采用CT引导下定位可以明显提高手术切除率^[1-4]^，目前用于CT引导下定位的材料主要分为液体材料和金属材料两大类，液体材料中最早用于CT引导下定位的是亚甲蓝，近年来国内学者纷纷报道了医用胶用于定位的方法及优势，但其本质上属于亚甲蓝定位法的升级版本^[3]^。金属材料中最早用于CT引导下定位的是Hook-wire，后期出现的微弹簧圈用于肺结节的定位可以视做Hook-wire的改良版本^[3, 4]^。

微弹簧圈定位的优点在于术中定位明确，有良好的组织相容性，对病灶组织微环境干扰小，且定位后距手术时间窗可以相对宽，是非常理想的定位材料，与Hook-wire定位对比，发生气胸、血胸、肺内出血的并发症机率较低^[5-10]^。本研究中微弹簧圈组发生气胸的比例为22.2%，大部分为极少量无症状的气胸，仅1例出现中等量气胸，分析原因，该患者存在肺内多发结节，反复多次穿刺定位后出现气胸量增加。微弹簧圈定位的另一缺点是存在脱落移位的风险，本研究中4例弹簧圈脱钩的病例出现在定位表浅结节（结节距离胸膜 < 1 cm）及穿刺入路与胸膜角度过小（< 30°）的情况，与相关文献报道相符^[8, 11]^，提示微弹簧圈定位对结节位置的要求更高，在本研究中体现微弹簧圈组选择定位结节的深度更深。此外部分微弹簧圈定位的病例中出现了圈尾盘于壁层胸膜及肌肉内，有1例术中探查胸腔广泛粘连，电钩分离过程导致部分圈尾残留在胸壁中，后行二次手术取出，因此弹簧圈尾的处理不当对手术存在一定的负面影响，也间接说明微弹簧圈定位技术要求更高。

近年来国内学者采用医用胶硬化剂进行定位，报道效果显著^[12-15]^，受此启发，我们从2017年7月开始采用硬化剂作为定位剂，所采用的聚桂醇注射液可用于血管瘤、脉管畸形、肝囊肿、肾囊肿、子宫肌瘤等多种疾病的硬化治疗，其主要成分为聚氧乙烯月桂醇醚，辅料为乙醇和水，其注射后可在靶区脉管内皮细胞表面的细胞脂膜相互作用，使内皮细胞脱落，形成血栓、管壁粘连，稳定性好^[16-18]^。在肺泡组织中应用后可在定位靶区形成明显的血肿样结节，术中易于探查（图 1）。CT影像上形成靶区团块和注射针道组成的“蒲公英”效应（图 2）。硬化剂定位最大的优点是其操作的简便性，平均穿刺操作时间（11.99±3.77）min，明显短于微弹簧圈定位时长（18.78±6.91）min，统计学上有显著性差异。学习曲线短，笔者观摩学习3例硬化剂定位后即开始独立定位。此外，聚桂醇注射液价格便宜，仅为微弹簧圈费用的1/4，有利于对人均住院费用和医用耗材的控制。与国内文献报道类似的是，本研究中硬化剂定位发生气胸、血胸及肺泡内出血等并发症的比例明显少于微弹簧圈组，对总体并发症的相关因素分析结果充分说明硬化剂的优势，但大部分国内学者报道的医用胶定位的最大缺点是存在刺激性干咳^[13-15]^，聚桂醇定位实际上也存在相同的缺点，但咳嗽持续的时间较短，且仅出现在注入药物的过程中，如控制好注射速度和量（不超过2 mL），咳嗽的症状能够进一步减轻，可能与聚桂醇同时属于醚类麻醉药，有轻微的局部麻醉的作用有关。采用硬化剂定位后一般应尽早开展手术，超过24 h以上会造成定位靶区组织尤其是微血管的严重破坏，本文中有2例患者定位40 h后实施手术，术中冰冻病理均提示组织充血水肿较重，对结节性质的读判造成一定的影响。

**1:**
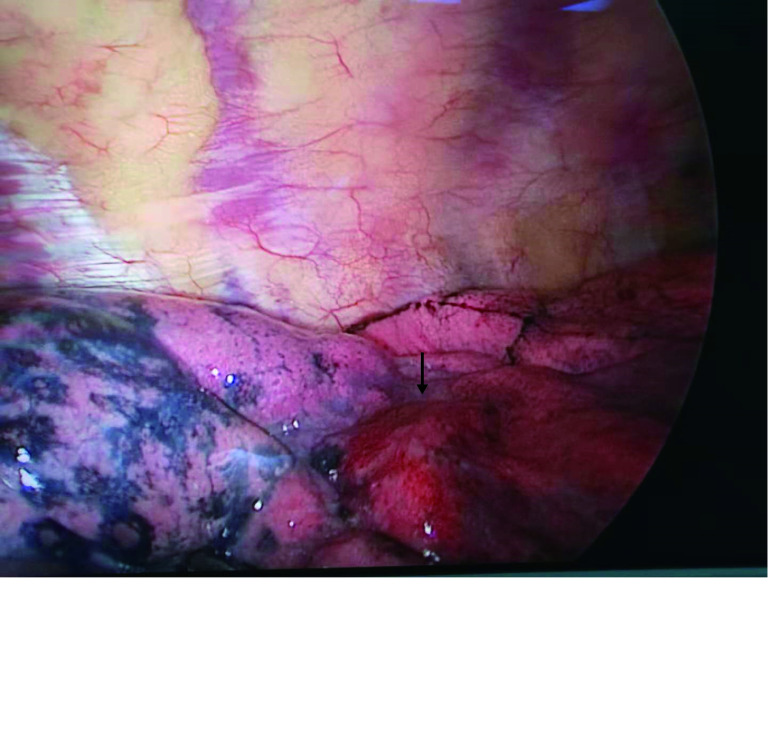
箭头所指为硬化剂定位形成的血肿样结节 The arrow indicate hematoma-like nodules that are localized by sclerosing agent

**2:**
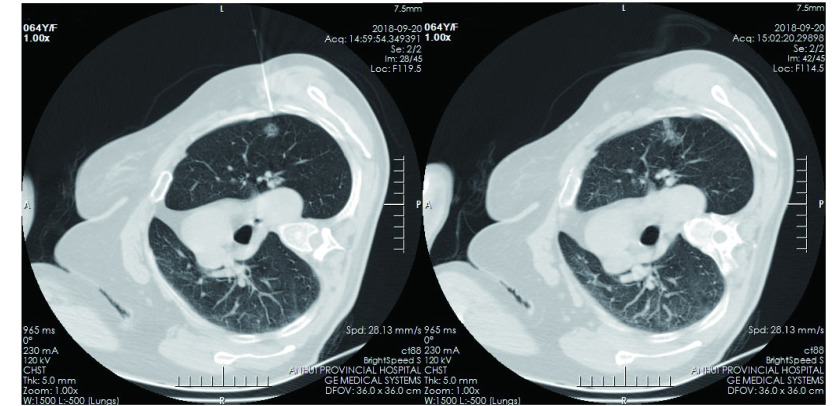
CT影像上形成靶区团块和注射针道组成的“蒲公英”效应 The "dandelion" effect is formed on CT image, which consists of target area mass and injection tract. CT: computed tomography.

在胸腔镜手术切除的方案上，SPN大部分采用亚肺叶切除，距离脏层胸膜1.5 cm以内的小结节，多采用楔形切除。位置更深的结节，为了保证足够的切除范围，必须采用肺段切除或肺叶切除，亚肺叶切除后如术中冰冻病理结果提示原位腺癌（adenocarcinoma *in situ*, AIS）或微浸润性腺癌（micro-infiltrating adenocarcinoma, MIA），一般不做严格的肺门纵隔淋巴结清扫。冰冻结果报道为浸润性腺癌者，需改做肺叶切除并行标准的肺门纵隔淋巴结清扫^[19]^。随着对肺小结节的认识逐步提高，手术指征的把控越加严格，恰巧两种定位方法应用于临床的先后有别（微弹簧圈早于硬化剂），造成两组病例病理类型中早期肺癌的比例呈上升趋势，而癌前病变的比例呈下降趋势，证明肺小结节的治疗理念正在逐步转变。于此同时，随着腔镜肺段技术的进步及应用指征的增宽，相当一部分小结节的术前定位价值正在变弱，本研究中实际诊断为肺多发病变者86例，有35例未行术前定位的结节同样完成了同期手术切除，但对于单发的外周小结节，楔形切除手术的优势依然明显^[20]^。术前定位技术使得肺内结节在肺脏层胸膜表面投影，实现可视化，但对萎陷后肺内结节深度的判断帮助有限，无论何种定位方式都无法准确的显示结节切除的下缘是否足够，我们在硬化剂组中尝试穿刺至结节下方注射一定剂量聚桂醇，使得靶区硬化范围更大，对术中评估切除范围起到了一定帮助，但穿刺的深度越深，发生气胸、血胸及肺泡内出血等并发症的概率越高^[6]^。如何才能更好的解决切缘的标示，仍是个亟待解决的问题。

综上所述，微弹簧圈与硬化剂均是良好的术前定位材料，聚桂醇硬化剂较之微弹簧圈定位失败率更低、并发症更少，定位时长更短，操作简便且费用低廉，值得推广。
